# Supplementary material for: Pramlintide: A Novel Therapeutic Approach for Osteosarcoma through Metabolic Reprogramming
Source: Cancers (Basel). 2022 Sep 2;14(17):4310. doi: 10.3390/cancers14174310 (PMC9454976; doi:10.3390/cancers14174310)

Figure S1

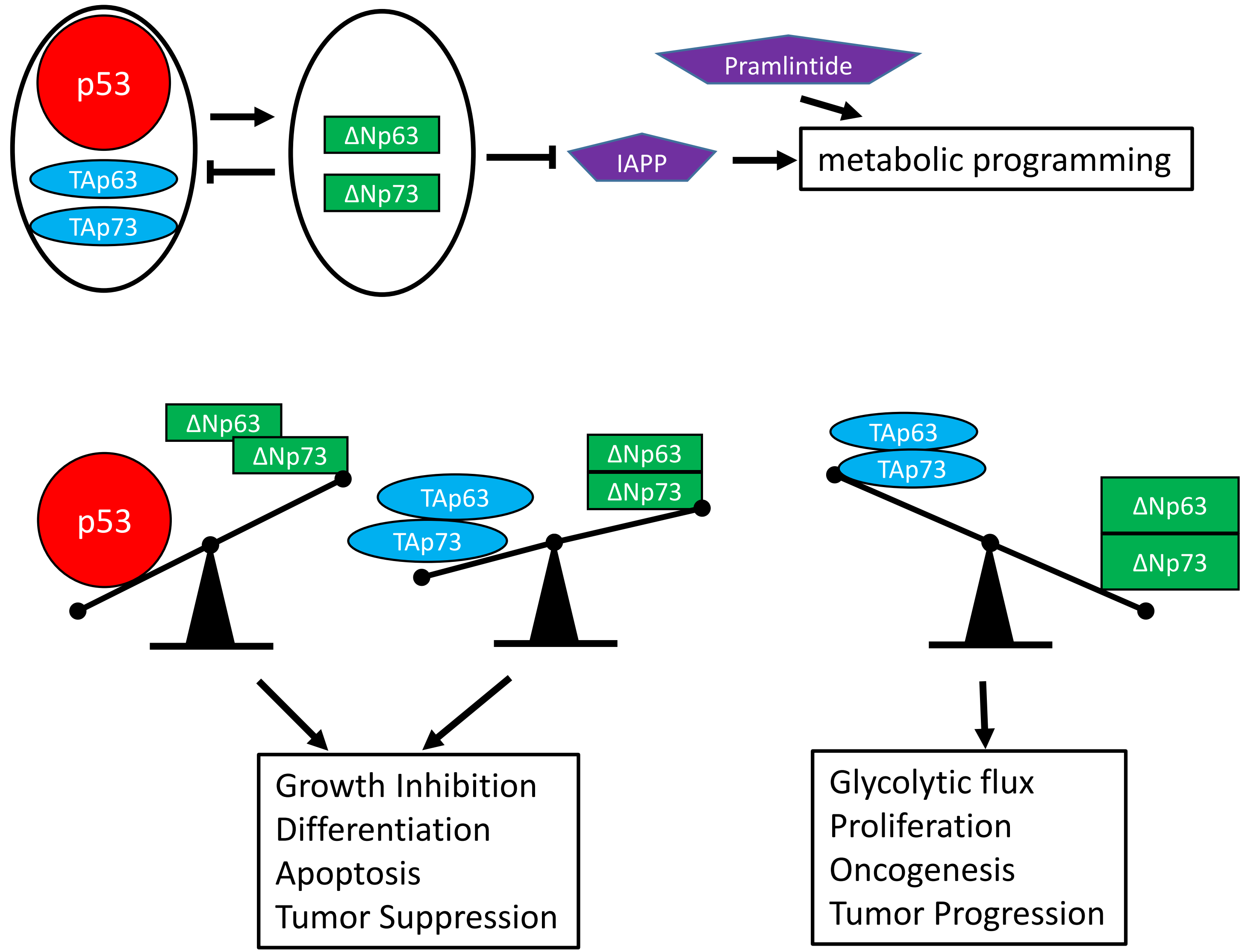

Figure S1. Balance of p53 family for cell control.

Figure S1

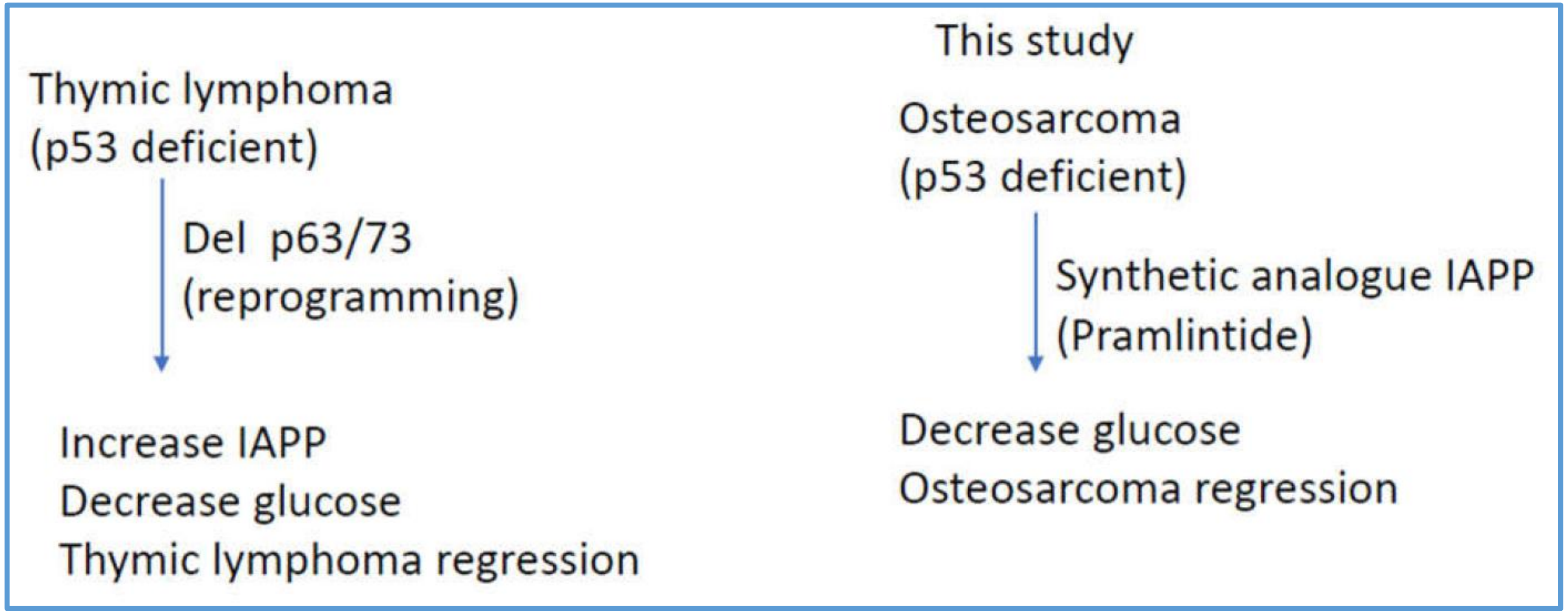

Figure S1. Balance of p53 family for cell control.

Figure S2

Ctrl placebo treatment | Pramlintide treatment

Before treatment

After 2 treatments

After 4 treatments

After 6 treatments

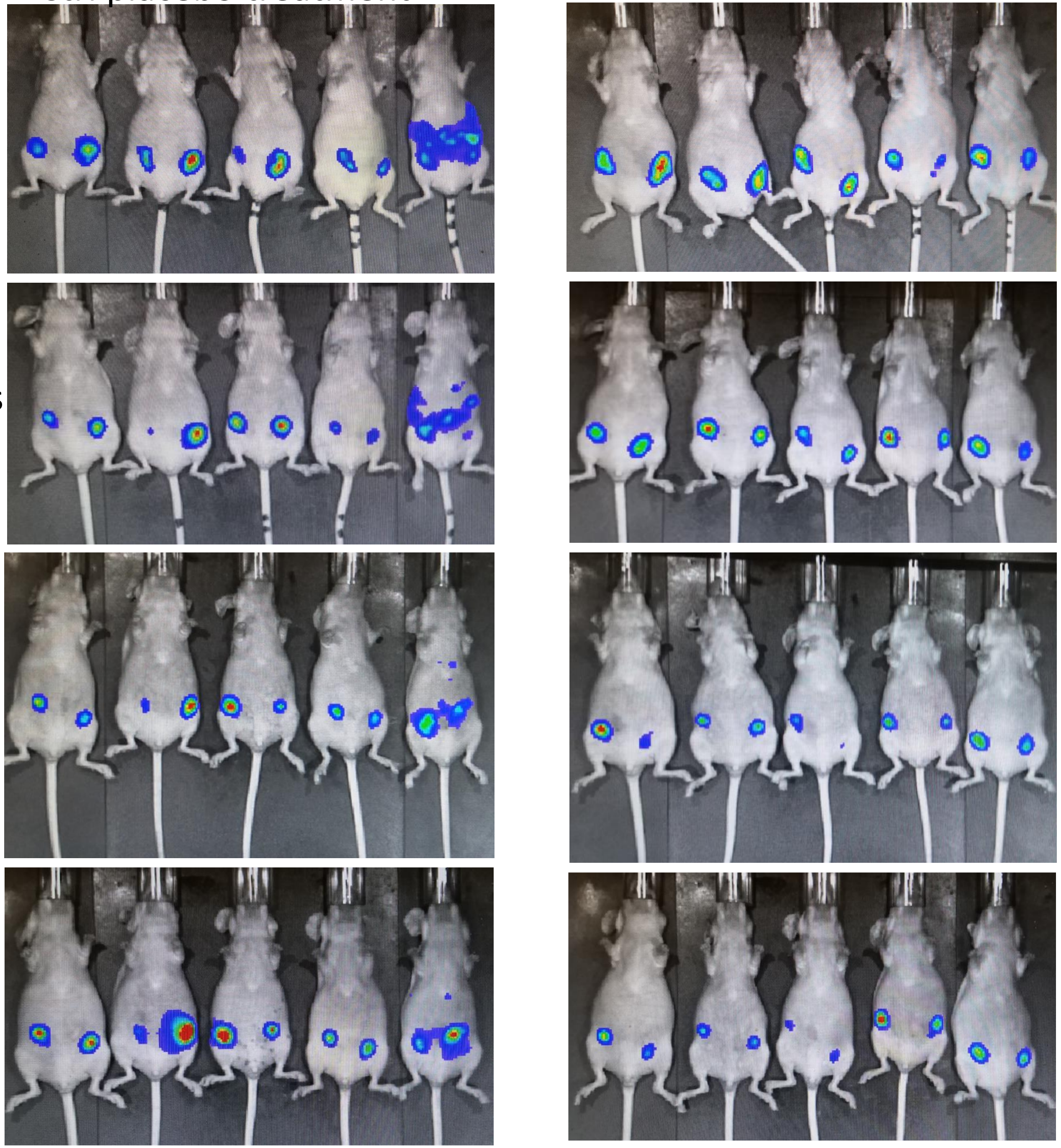

Figure S3 A, B

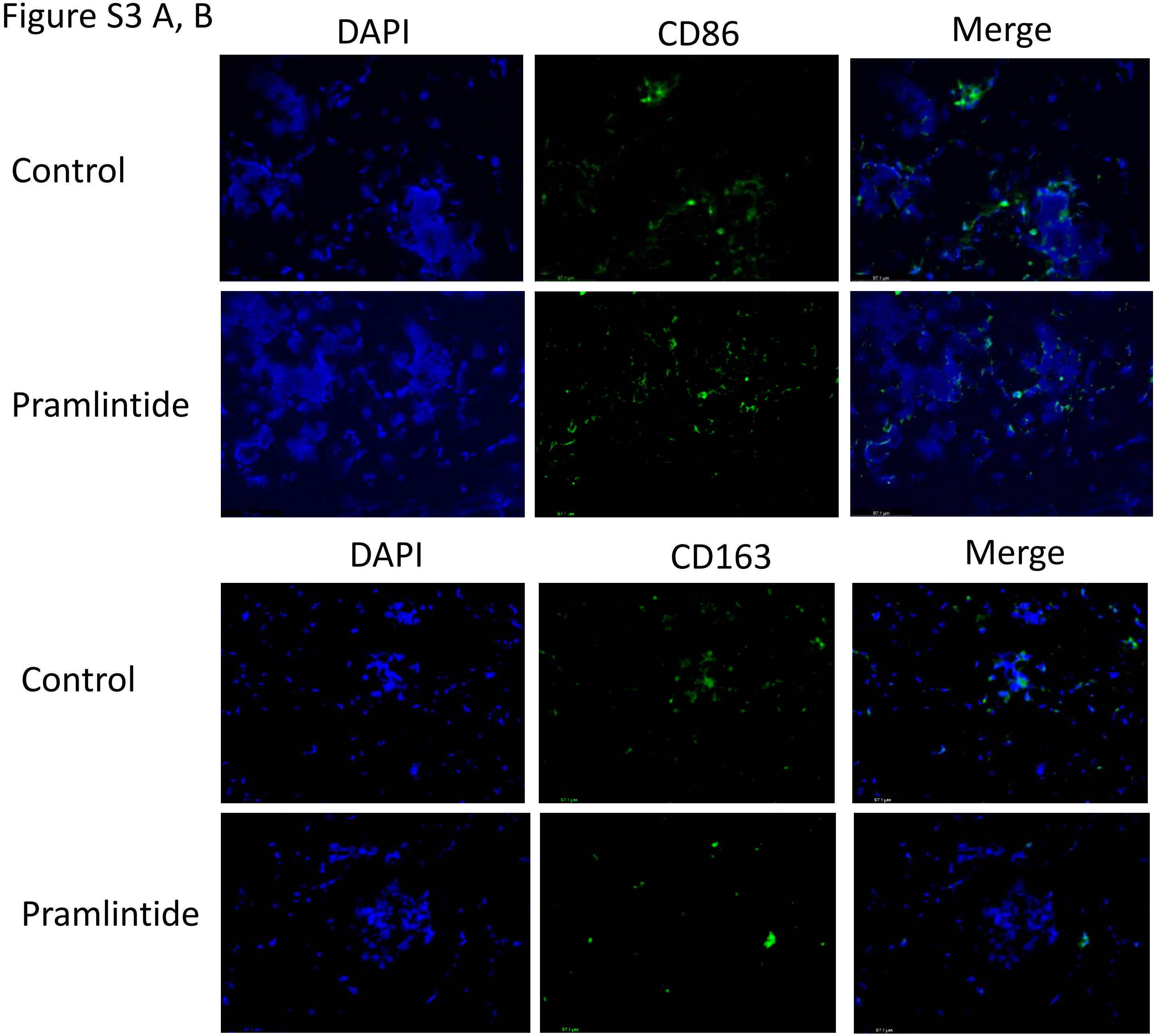

M1(CD86) and M2(CD163) staining

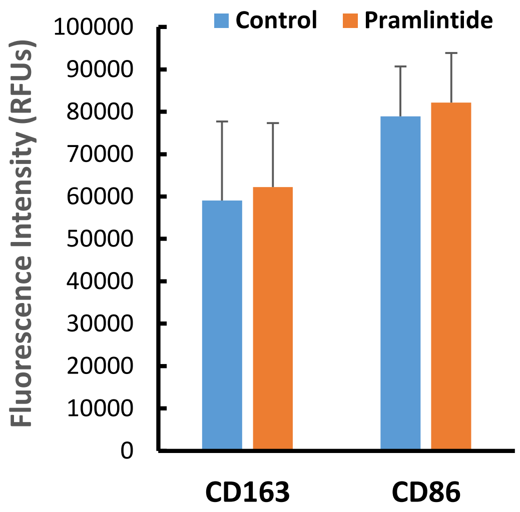

Supplement: Supplementary file 1 [file cancers-14-04310-s001.zip › cancers-1843408-Figure S1-S3.pdf]
