# Supplementary material for: Pramlintide: A Novel Therapeutic Approach for Osteosarcoma through Metabolic Reprogramming
Source: Cancers (Basel). 2022 Sep 2;14(17):4310. doi: 10.3390/cancers14174310 (PMC9454976; doi:10.3390/cancers14174310)
Supplement: Supplementary file 1 [file cancers-14-04310-s001.zip › cancers-1843408-File S1.pdf]

Figure 1A

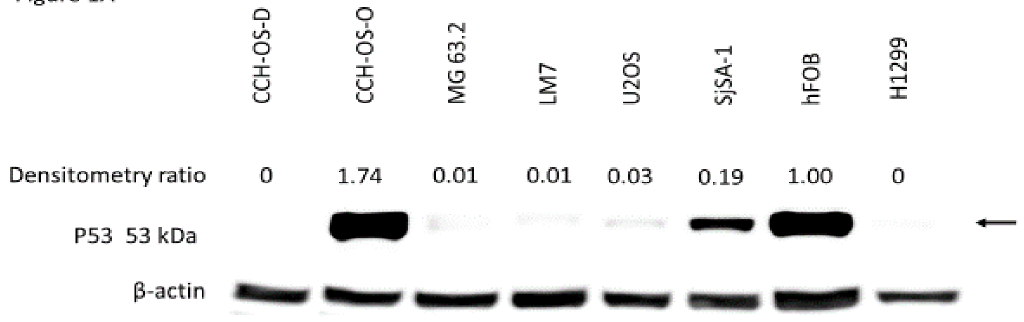
CCH-OS-D
CCH-OS-O
MG 63.2
LM7
U2OS
SJSA-1
hFOB
H1299
Densitometry ratio
0
1.74
0.01
0.01
0.03
0.19
1.00
0
P53 53 kDa
β-actin

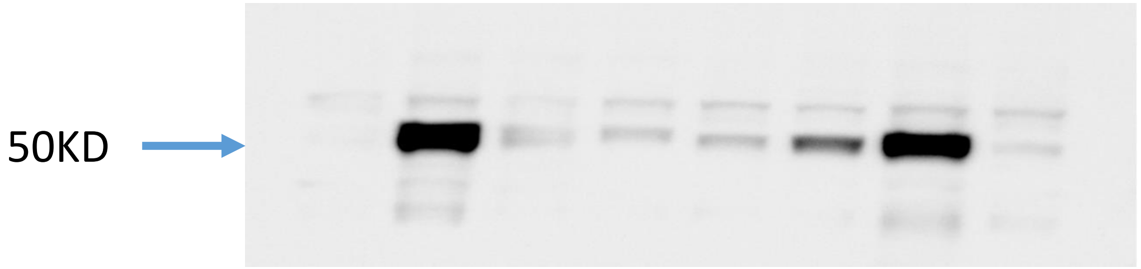
50KD

Figure 1B

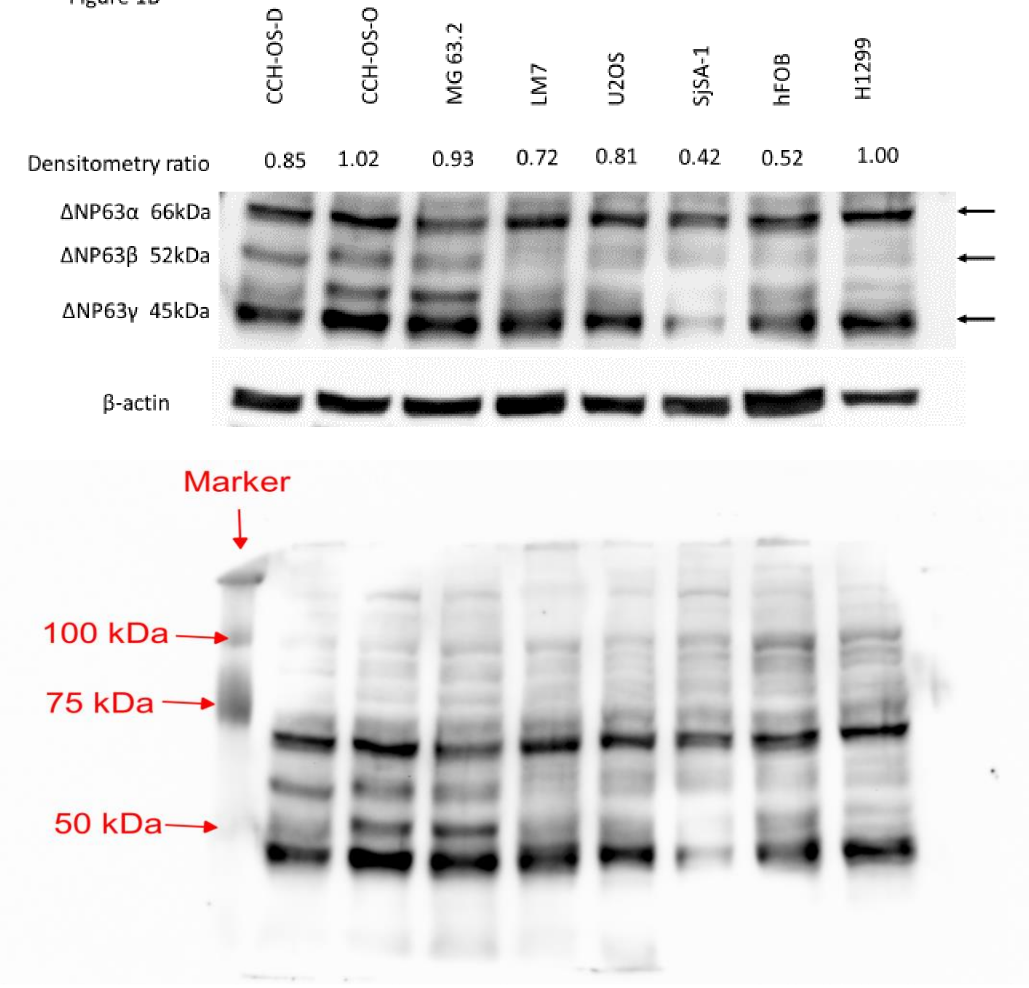
CCH-OS-D
CCH-OS-O
MG 63.2
LM7
U2OS
SjSA-1
hFOB
H1299
Densitometry ratio
0.85
1.02
0.93
0.72
0.81
0.42
0.52
1.00
ΔNP63α 66kDa
ΔNP63β 52kDa
ΔNP63γ 45kDa
β-actin
Marker
100 kDa
75 kDa
50 kDa

Figure 1C

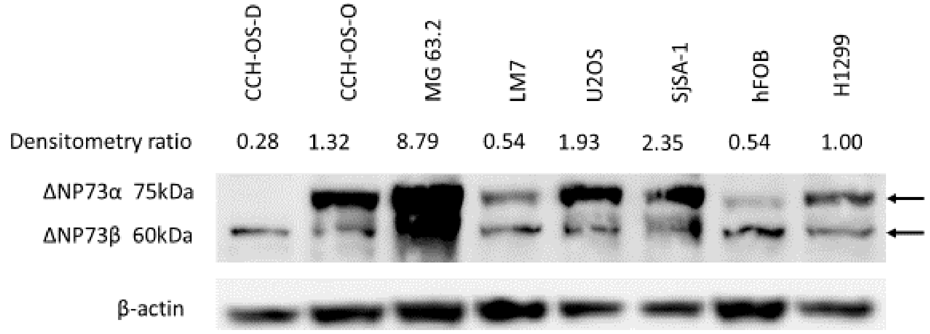
CCH-OS-D
CCH-OS-O
MG 63.2
LM7
U2OS
SJSA-1
hFOB
H1299
Densitometry ratio
0.28
1.32
8.79
0.54
1.93
2.35
0.54
1.00
ΔNP73α 75kDa
ΔNP73β 60kDa
β-actin

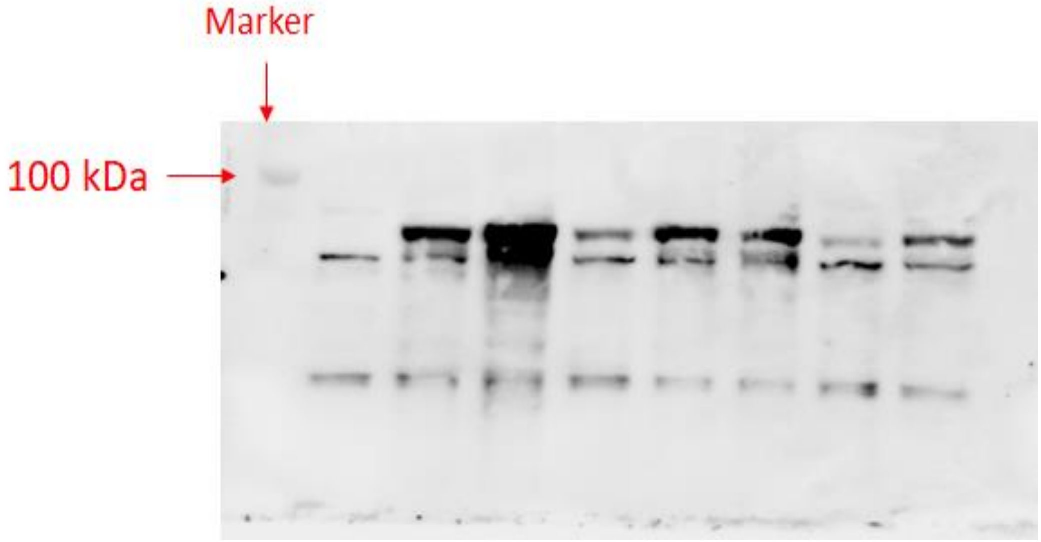
Marker
100 kDa

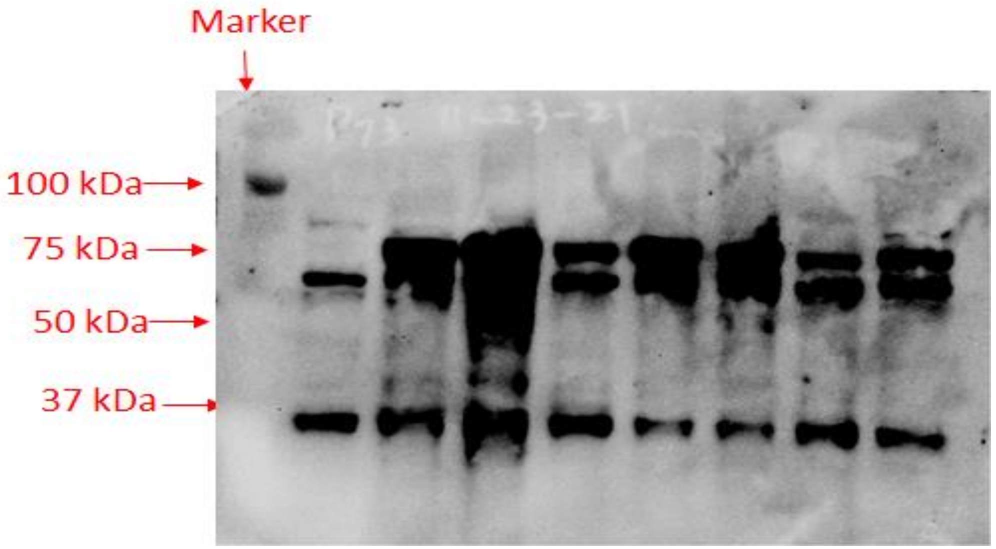
Marker
100 kDa
75 kDa
50 kDa
37 kDa

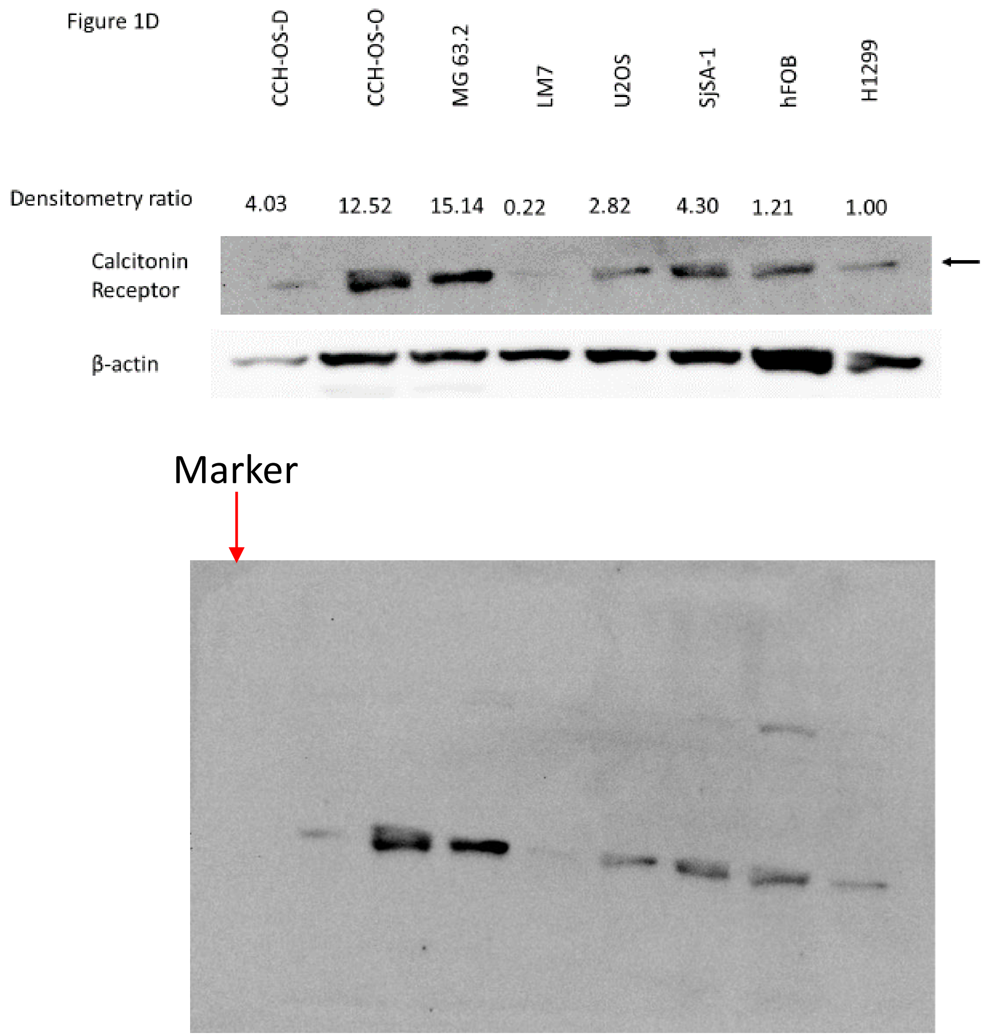
Figure 1D
CCH-OS-D
CCH-OS-O
MG 63.2
LM7
U2OS
SJSA-1
hFOB
H1299
Densitometry ratio
4.03
12.52
15.14
0.22
2.82
4.30
1.21
1.00
Calcitonin Receptor
β-actin
Marker

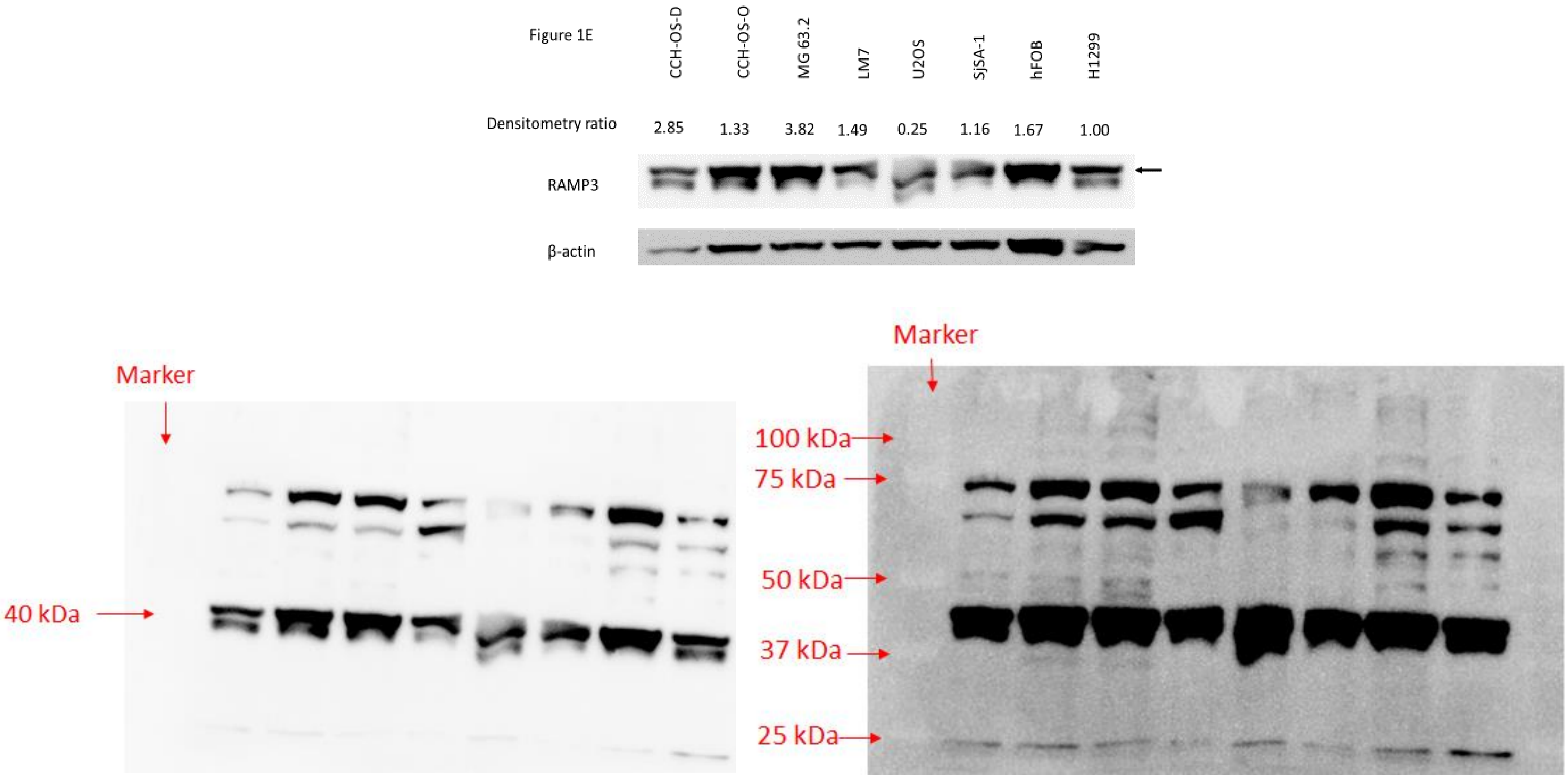
Figure 1E
CCH-OS-D
CCH-OS-O
MG 63.2
LM7
U2OS
SjSA-1
hFOB
H1299
Densitometry ratio
2.85
1.33
3.82
1.49
0.25
1.16
1.67
1.00
RAMP3
β-actin
Marker
40 kDa
Marker
100 kDa
75 kDa
50 kDa
37 kDa
25 kDa

Figure 1F

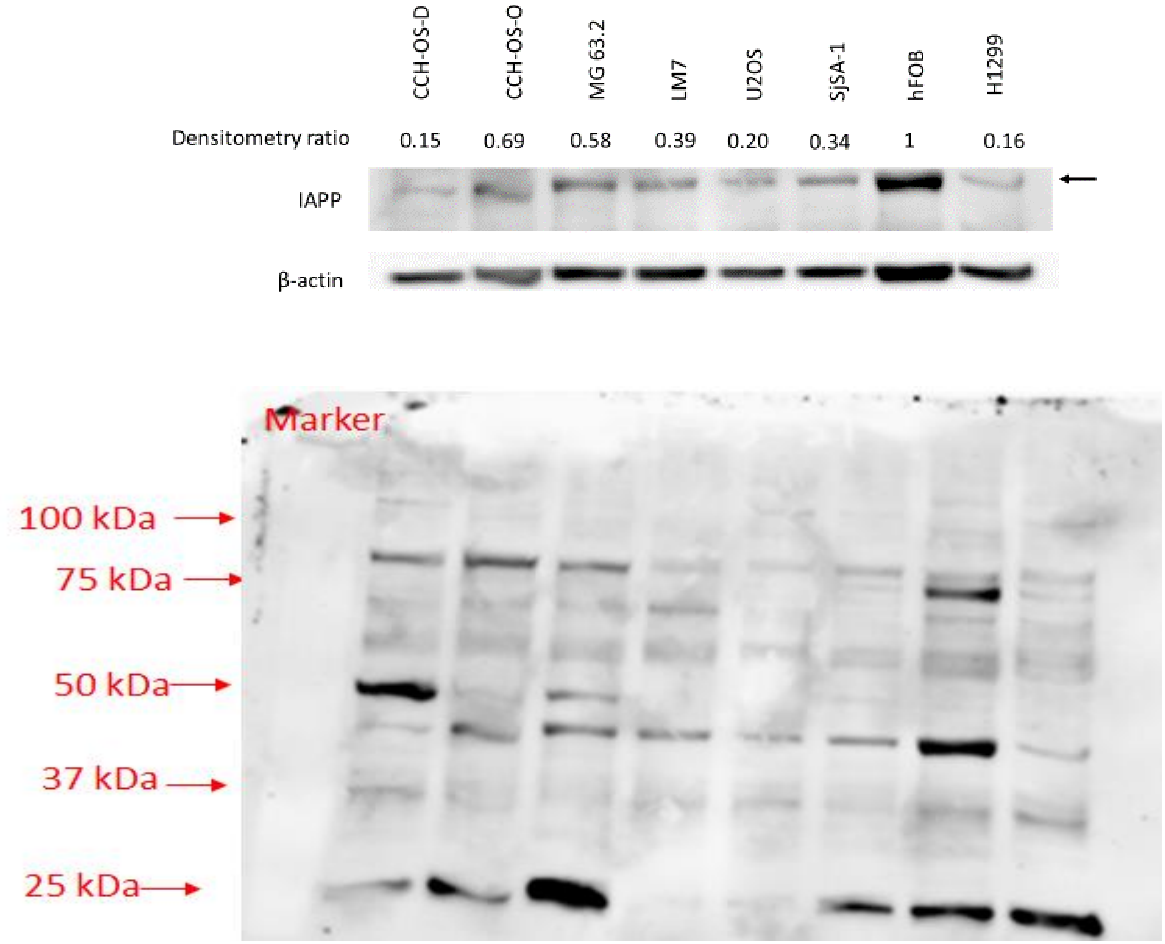
CCH-OS-D
CCH-OS-O
MG 63.2
LM7
U2OS
SjSA-1
hFOB
H1299
Densitometry ratio
0.15
0.69
0.58
0.39
0.20
0.34
1
0.16
IAPP
β-actin
Marker
100 kDa
75 kDa
50 kDa
37 kDa
25 kDa

Figure 4A

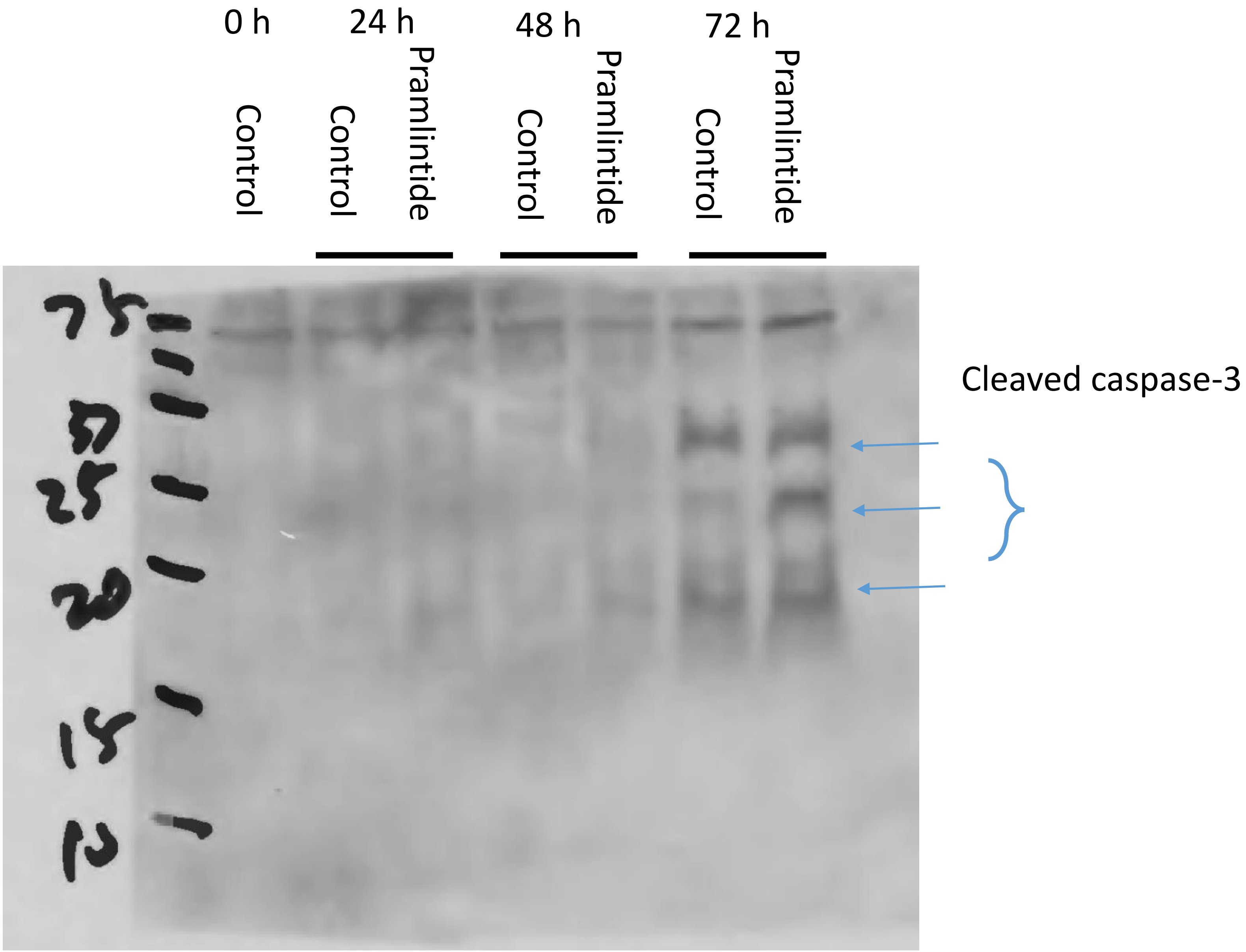
0 h
24 h
48 h
72 h
Control
Control
Pramlintide
Control
Pramlintide
Control
Pramlintide
Cleaved caspase-3

Figure 4B

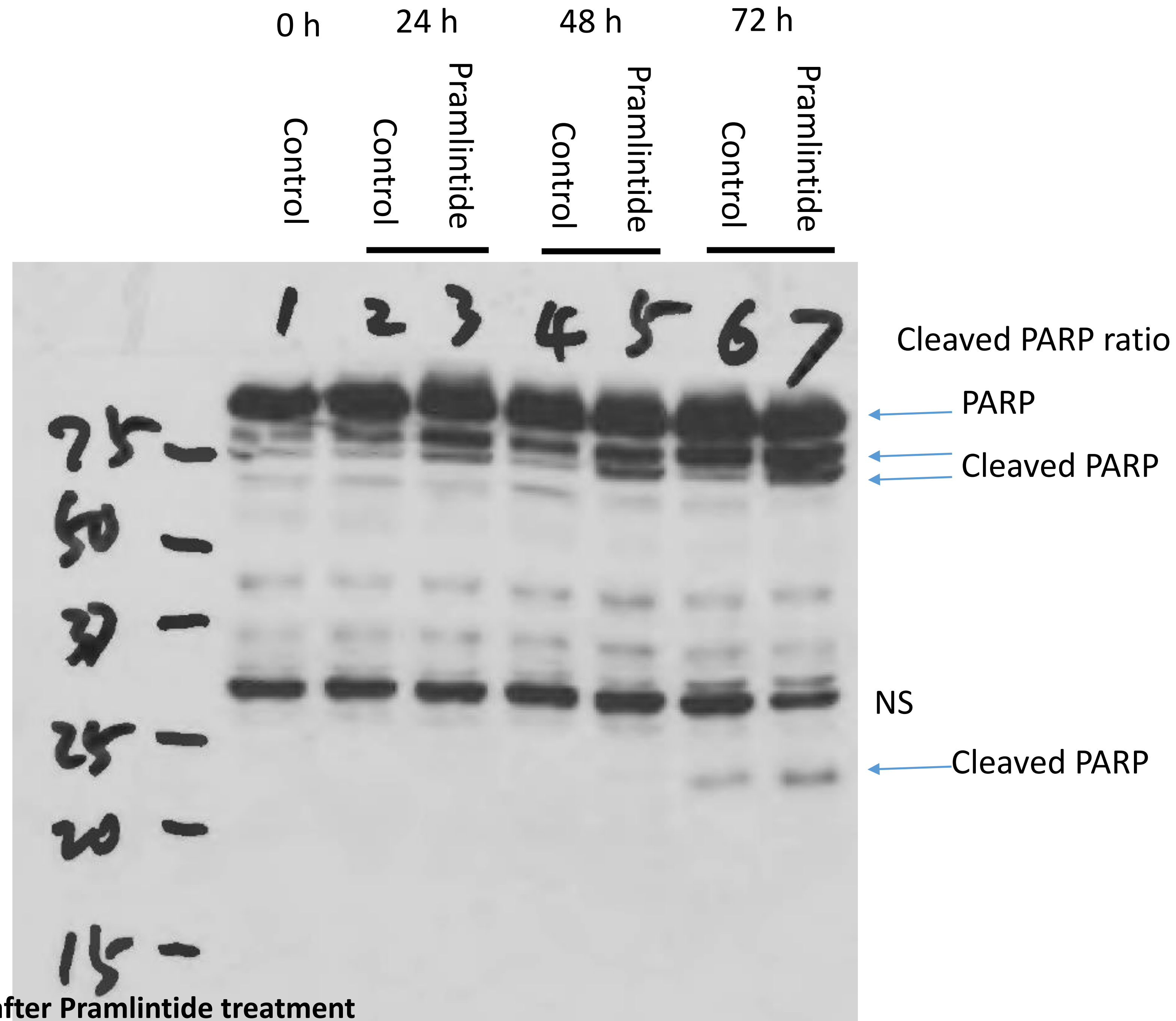

Increased Cleaved PARP in CCH-OSD after Pramlintide treatment

Figure 4C, D, E

C

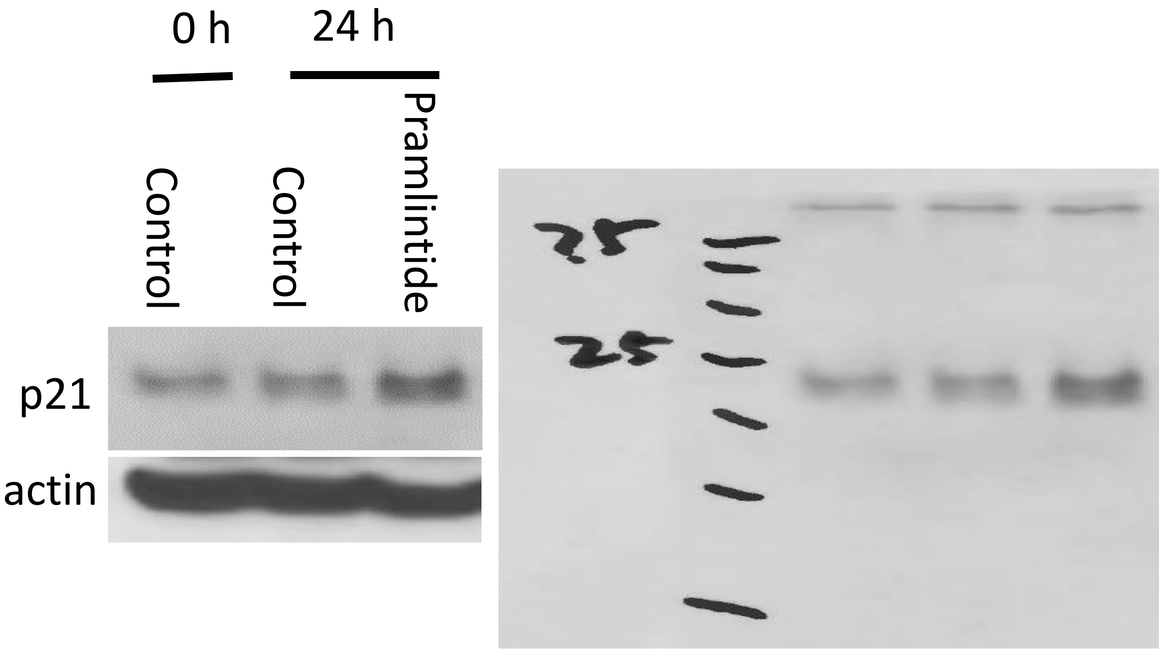

Figure 4C, D, E Increased p21, p27 but not cyclin D3 in CCH-OSD after Pramlintide treatment

Figure 4C, D, E

D

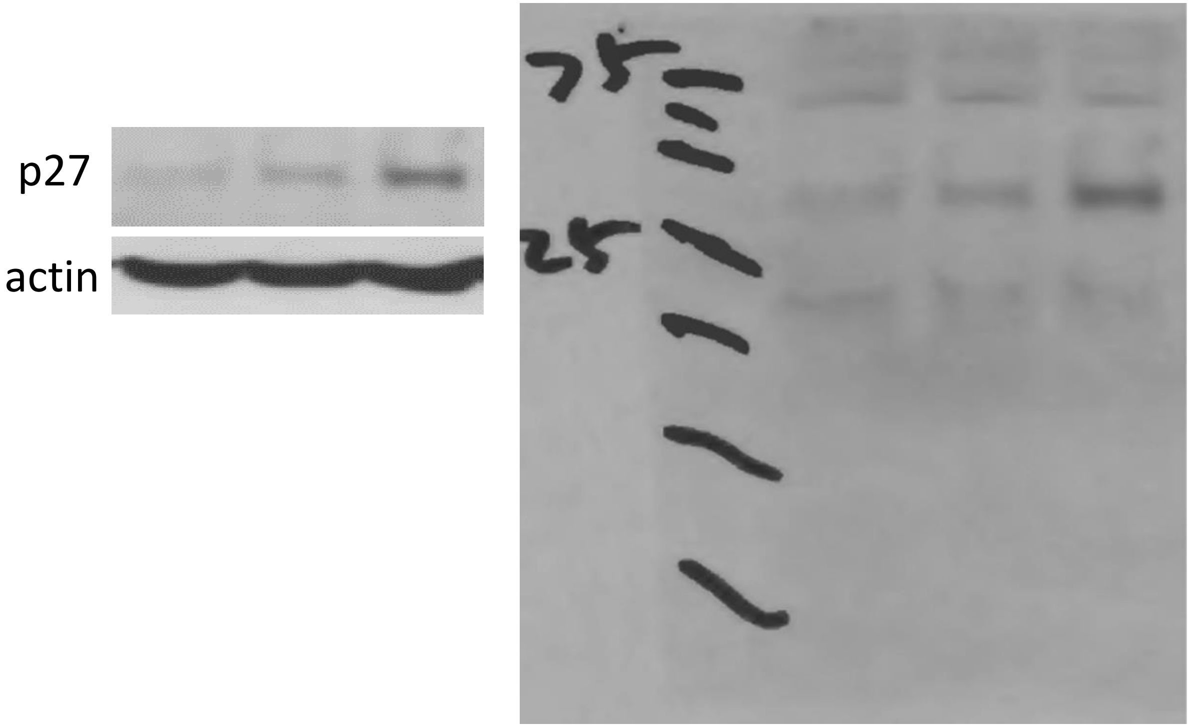

Figure 4C, D, E Increased p21, p27 but not cyclin D3 in CCH-OSD after Pramlintide treatment

Figure 4C, D, E

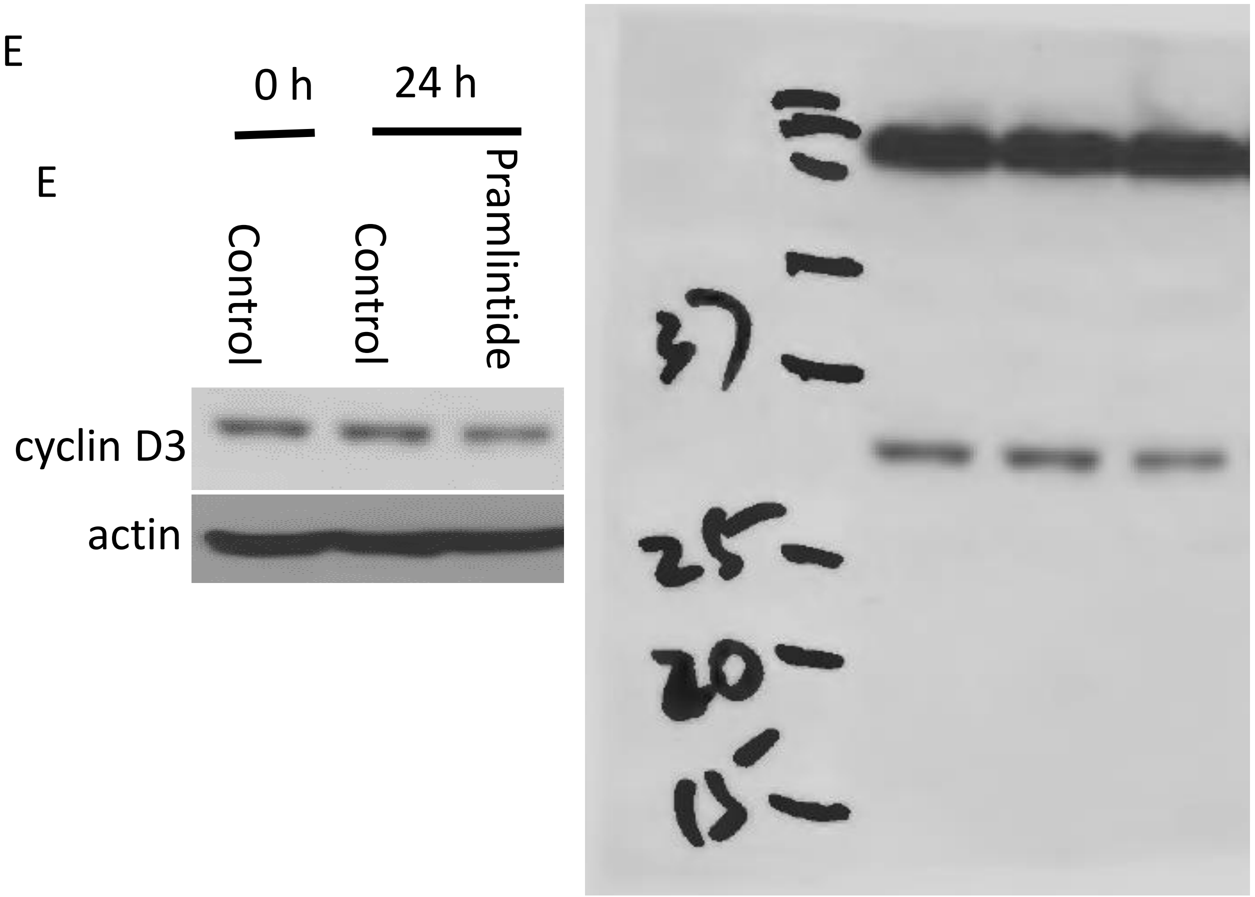

Pramlintide 20ug/ml

Figure 4C, D, E Increased p21, p27 but not cyclin D3 in CCH-OSD after Pramlintide treatment
